# Heavy Metal Accumulation is Associated with Molecular and Pathological Perturbations in Liver of *Variola louti* from the Jeddah Coast of Red Sea

**DOI:** 10.3390/ijerph13030342

**Published:** 2016-03-21

**Authors:** Saleh A. Mohamed, Mohamed F. Elshal, Taha A. Kumosani, Ahmad O. Mal, Youssri M. Ahmed, Yaaser Q. Almulaiky, Amer H. Asseri, Mazin A. Zamzami

**Affiliations:** 1 Biochemistry Department, Faculty of Science; Production of Bioproducts for Industrial Applications Research Group and Experimental Biochemistry Unit, King Fahd Medical Research Center King Abdulaziz University, Jeddah 21589, Saudi Arabia; melshal2002@yahoo.com (M.F.E.); t.kumosani@yahoo.com (T.A.K.); youssri01@yahoo.com (Y.M.A.); yaser_almoliki@yahoo.com (Y.Q.A.); ahasseri@kau.edu.sa (A.H.A.); mzamzami@kau.edu.sa (M.A.Z.); 2 Marine Biology Department, Faculty of Marine Sciences, King Abdulaziz University, Jeddah 21589, Saudi Arabia; aomal@kau.edu.sa

**Keywords:** aquatic pollution, heavy metals, apoptosis, DNA damage, fibrosis, cytotoxicity

## Abstract

Large amounts of waste water are discharged daily from the Jeddah Metropolitan Area into the Red Sea. Sewage draining into the Red Sea causes widespread chemical pollution that is toxic to aquatic ecosystems. The objective of this study was to investigate the extent of pollution and assess the presence of heavy metals in fish tissue and study their association with biological and biochemical alterations. The average concentrations of heavy metals found in hepatic tissues of *Variola louti* fish from the polluted area, namely Cd, Cr, Cu, Fe and Zn, were 1.74, 9.69, 47.48, 4020.01 and 229.47 µg/g liver, respectively, that were significantly higher than that of samples taken from reference area (0.24, 1.98, 20.12, 721.93, 129.21 µg/g liver, respectively). The fold change of heavy metals in fish from the polluted area with respect of that of the reference area followed the order Cd > Fe > Cr > Cu > Zn. Analysis of nuclear DNA revealed that hepatic tissues of fish samples from the polluted area showed a significant increase in apoptotic cells as detected by flow cytometry and formation DNA-ladder. In addition, hepatic sections from polluted area fishes showed more fibrotic changes and collagen deposition by hematoxylin-eosin staining and Masson’s trichrome staining, respectively, compared to samples taken from the reference area. Moreover, the electrophoretic patterns of proteins of liver of fishes caught at the polluted area showed different patterns of proteins from that of the reference with bands at 42, 130 and 140 kDa, which is in a good agreement with the molecular weight of collagen type III. In conclusion, there were significant changes in the tissues of fishes in the polluted area at the cellular and the molecular levels that may be associated with an accumulation of heavy metals. Assessment of fishes as a sensitive biomonitor for the pollution of surface waters that may affect general health of human and wild life is conceivable.

## 1. Introduction

A daily load of 100,000 m^3^ per day of sewage sludge are discharged in the Jeddah Metropolitan Area of the Red Sea which significantly reduces water quality, raises the ecological risk to human health [1], and increases generalized mortality of aquatic life [2]. Pollutants from oil spillages may be directly responsible for deaths of large numbers of aquatic animals, whereas sewage sludge pollutants contains organic material, e.g., faecal debris, which cause decreases in water quality, oxygen depletion and changes in pH values leading to an increase in microbial populations and greater susceptibility of aquatic organisms to attack by pathogens [3,4,5]. In addition to organic contaminants, heavy metals are considered one of the main groups of pollutants that enter aquatic systems via anthropogenic activities and/or atmospheric deposition. Large amounts of heavy metals contaminating aquatic systems are accumulated and biomagnified through water, sediment, and the aquatic food chain, resulting in devastating impact on the ecological balance of the recipient environment and on a variety of aquatic organisms [6,7]. Excess amounts of these metals entering the aquatic ecosystem may pollute the environment and also affect the food chain and ultimately pose serious human health risks [8].

Ionic forms of metals are generally more toxic due to their ability to react with other ions catalysing the formation of toxic compounds such as oxyradicals. Oxyradicals, which are formed as a result of electron transfer reactions, are of considerable importance in both animals and plants. Some oxyradicals, such as superoxide anion (O_2_^–^) and the hydroxyl radical (OH^–^), can cause serious cellular damage [9,10]. Research has indicated that heavy metals may directly affect cellular organelles and components such as nucleic and mitochondrial DNA, and may inhibit some enzymes involved in DNA damage repair causing DNA damage and conformational changes that may lead to cell cycle modulation, and apoptosis [11].

Metals are natural elements of the environment that are found in varying levels in ground and surface waters, and are classified into two types; essential and non-essential elements. Essential elements such as copper (Cu), iron (Fe), manganese (Mn), nickel (Ni) and zinc (Zn) are required for various biochemical and physiological functions, while other metals including cadmium (Cd), chromium (Cr), mercury (Hg), lead (Pb), arsenic (As), and antimony (Sb) are non-essential and play no significant biological role. Living organisms require trace amounts of essential heavy metals, but these can become toxic at high doses [12,13], whereas, non-essential metals such as cadmium [14], chromium [15], copper [16], iron [17] and zinc [18] are highly toxic and can exert their toxicity at low doses, and consequently these five elements rank among the priority metals that are of great public health significance. These metals may accumulate in the food chain and pose carcinogenic and other adverse risks to human health due to bioaccumulation over time. Research has indicated that environmental exposure to heavy metals increases the risk for developing cancers, diabetes, kidney failure and damage to the nervous system [8,19,20]. However, connections must be established between the external levels of exposure of these elements, internal levels of tissue contamination and their adverse effects.

As fish are constantly to exposed pollutants in contaminated water, they could be used as excellent biological markers of heavy metals in aquatic ecosystem [21]. In addition, the liver can be regarded as the body’s detoxification organ and hence a target organ of various xenobiotic substances. Therefore, we aimed to investigate the levels of heavy metals which contaminate aquatic ecosystems, and identify their adverse effects in liver tissues, DNA damage and new stress proteins of *Variola louti* as sentinel species in polluted sites of the Jeddah coastal sea water and comparison to fishes from a pristine reference site.

## 2. Materials and Methods

### 2.1. Sample Collection and Handling

Fish samples of the type *Variola louti* were collected during four different times per year in 2014 and 2015 from two areas of Jeddah’s southern Red Sea shore: (i) a polluted area at Al-Badhae, where abundant of sewage was received; (ii) a reference area at Shuarrah. The annual temperature of the sea water in Jeddah city ranged from 25 to 29 °C. Only fish over 20 cm in length were sampled. Within 2 h the catch were transferred to 50-L tanks filled with aerated seawater and transported to the laboratory where the individuals were dissected during the same day. The dissected liver tissues were frozen and stored at −80 °C until biomarker analyses. The fishing was conducting according to (Decree No. 21911, 7 November 1988) of Ministry of Agriculture, Kingdom of Saudi Arabia. Fish sampling was authorized by Royal Coast Guard of Kingdom of Saudi Arabia (Decree No. 2, 3 February 1990). King Abdulaziz University abides by Royal Decree No. M/59, 24 August 2010 entitled “Research Ethics for Handling of Living Animals”.

### 2.2. Preparation of Tissue Homogenates

A 200–300 mg slice of liver was homogenized in cold homogenizing buffer (100 mM K_2_HPO_4_/KH_2_PO_4_, 150 mM KCl, 1 mM dithiothreitol, 1 mM EDTA, pH 7.5) at a ratio of 1:5 (weight:volume). The homogenates were centrifuged at 12,000 g for 20 min at 4 °C. The supernatant were collected and stored at −80 °C until use.

### 2.3. Trace Metals Determination

Trace metals determination was performed as previously described by Santos *et al*. [6] with minor modifications. Briefly, each one g of liver tissue sample was heated in a porcelain crucible at 600 °C for 16 h. Ten mL of deionized water was added and filtered through Whatman No. 1 filter paper. The filtrate was analyzed for levels of Cd, Cu, Cr, Fe and Zn by inductively coupled plasma atomic emission spectrometry (ICPE-9000; Shimadzu Scientific Instruments Inc., Kyoto, Japan). 

### 2.4. DNA Flow Cytometry Analysis

DNA-cell cycle distribution was investigated by flow cytometry after staining the cells with propidium iodide (PI). Small pieces of the liver were minced and disaggregated mechanically using nylon mesh into cell suspension. Cells were washed with phosphate-buffer saline (PBS) and centrifuged at 100 g for 5 min, the cell pellet was suspended with 70% ethanol and kept at −20 °C for 12 h. Then, the cells were washed with cold PBS and suspended in PBS containing 20 μg/mL PI, 0.2 mg/mL RNase A and 0.1% Triton X-100 at 4 °C for 12 h. The stained cells were then analyzed by a FACSCalibur System flow cytometer (Becton Dickinson Biosciences, San Jose, CA, USA). To analyze apoptosis, hypodiploid DNA (Sub-G1) populations were assayed using a FACSCalibur flow cytometer with Cell Quest software (Becton Dickinson) as previously described [22]. For all assays, 10,000 events were counted and the percentage of apoptotic cells was determined using ModFit software.

### 2.5. DNA-Ladder Fragmentation Assay

The Apoptotic-LADDER assay requires the preparation of nucleosomal DNA to investigate the presence of DNA fragmentations. The procedure does not involve the use of toxic phenol. The protocol involves cell lysis; removal of cellular debris and subsequent precipitation of nucleosomal DNA. Nucleosomal DNA is detected after standard 1.8% agarose gel electrophoresis. Single cell suspension from the liver samples was prepared and cells were pelleted at 2000× g for 20 s and resuspended in 100 µL of phosphate-buffered saline. DNA was extracted by the method of Hirt [23], with minor modifications. Briefly, the cell suspension was lysed by adding 400 µL of TE buffer (10 mM Tris-HCl buffer, pH 7.4 and 10 mM EDTA) containing 0.6% sodium lauryl sulfate. The cell lysate was gently mixed with 125 µL of 5 M NaCl and kept at 4 °C for two hours. The mixture was centrifuged at 14,000× *g* for 30 min, and the chromatin pellet was then removed. After treatment with RNase and proteinase K, DNA in the supernatant was precipitated with ethyl alcohol and resuspended in TE buffer. Samples were analyzed for a nucleosomal DNA ladder by electrophoresis on a 1.8% agarose gel. DNA extracts were quantified using NanoDrop ND-1000 (Thermo Fisher Scientific Inc., Wilmington, DE, USA) as previously descriped [24]. Agarose gel electrophoresis was used to detect DNA fragments after DNA extraction. Briefly, 1.8% agarose gel was prepared and 35 µL ethidium bromide was added. The gel was then placed into electrophoresis tank containing sufficient TAE buffer and three µL DNA sample mixed with 2 µL loading buffer was carefully loaded into wells on the gel. Appropriate molecular weight marker (1 kb ladder marker) (Jena Bioscience, #M-217, Jena, Germany) was also included in the same gel. The power supply was connected and adjusted to 100 volts for 60 min.

### 2.6. SDS-Polyacrylamide Gel Electrophoresis

Electrophoresis under denaturing conditions was performed in 10% (w/v) acrylamide slab gel according to the method of Laemmli [25] using a Tris-glycine buffer, pH 8.3. Protein bands were located by staining with Coomassie Brilliant Blue R-250.

### 2.7. Light Microscopy

Hepatic tissues from all fishes were fixed in 10% neutral buffered formaldehyde solution (pH 7.0). After fixation was complete (minimum time: 18–24 h), the tissues were processed by a routine histological method and stained by hematoxylin and eosin (H&E) as previously described [26]. Masson’s trichrome stain was applied to demonstrate fibrotic tissue. The intensity of Masson’s trichrome blue color staining depended on the content of collagen fibers in the investigated tissue [27]. In the Masson trichrome stained sections, the collagen quantitative analysis in liver was performed in three non-serial sections per fish, totaling 15 sections per group. The sections were analyzed using a BX51 microscope (Olympus, Melville, NY, USA) equipped with filters to provide circularly polarized illumination. All image acquisition parameters and the intensity of acquisition illumination were standardized by adjusting only the microscope condenser aperture. The images were obtained with an x40 objective lens, recorded on a digital camera (DP-71, Olympus, Melville, NY, USA) and analyzed using ImageJ^®^ image analysis software (http://rsbweb.nih.gov/ij/webcite). The analysis methodology was adapted from a method on the NIH website used to quantify stained liver tissue using a thresholding algorithm (http://rsbweb.nih.gov/ij/docs/examples/stained-sections/index.html) with minor modification. Briefly, Each image was converted from a RGB color image to a RGB stack (grayscale) and analyzed in the red channel. We noted three areas of interest within the grayscale image: a lighter gray region representative of stained cytosol (pink in color image), a significantly dark gray region representative of the stained collagen (blue in color image), and a white region indicative of dead space within in the liver section. Grayscale image was used, then the red-stained collagen was isolated using thresholding function. After that, the thresholded area was measured and results were reported as the percentage of collagen area per image as previously described [28].

### 2.8. Statistical Analysis

The statistical analyses were performed by a one-way ANOVA using Bonferroni’s post hoc test and the Student’s *t*-test. Levene’s test of homogeneity of variances was used to assess data distribution. The results were expressed as means ± S.D. Differences were considered significant when *p* < 0.05.

## 3. Results Discussion

**Figure 1:**
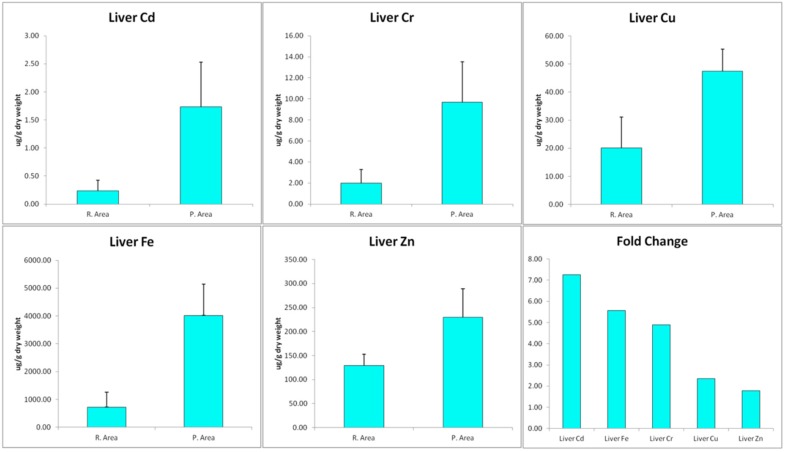
Concentrations of Cd, Cr, Cu, Fe and Zn in hepatic tissues (µg/g) of *Variola louti*.

In the last two decades, the rate of urbanization and industrialization has increased in Saudi Arabia. Besides the many problems associated with such social changes, pollution is considered to be a major concern for the health of the nation. Among the numerous types of environmental pollutions that threaten human health, contamination of surface waters and the surrounding aquatic ecosystem appear to be a growing threat that requires immediate attention and action. Research has indicated that there is a good correlation between the presence of pollutants in fish and the levels in the surrounding aquatic environment. For example, heavy metals in the tissues of chub (*Squalius cephalus*) and roach (*Rutilus rutilus*) obtained from the polluted waters around Czech Republic [29], *Tilapia* sp. and *Chrysichthys* sp. from the Ogun river of Nigeria [30] and Sava river at Zagreb [31]. In the present study, there was a significant increase (*p* < 0.001) in the content of Cd, Cr, Cu, Fe and Zn in the liver of fishes from polluted area in comparison to that from the reference area (1.74, 9.69, 47.48, 4020.01 and 229.47 *versus* 0.24, 1.98, 20.12, 721.93, 129.21 µg/g liver, respectively). The fold increase of heavy metals in fish from polluted area with respect of that of reference area followed the order Cd > Fe > Cr > Cu > Zn (Figure 1).

**Figure 2:**
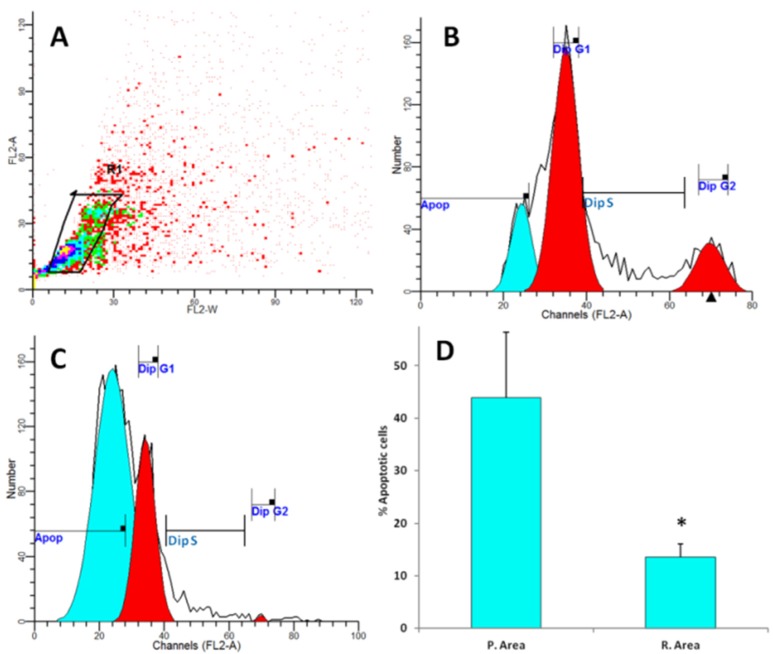
Representative flow cytometry histograms demonstrating the cell cycle phases as determined by propidium iodide staining. (**A**) gating out doublet events on the basis of FL-2A *versus* FL-2W; (**B**,**C**) cell cycle phases of hepatic tissues from reference and polluted area respectively; (**D**) the average of apoptotic cells percentage. Values represent the means ± SD, * *p* < 0.05 (Student *t*-test).

**Figure 3:**
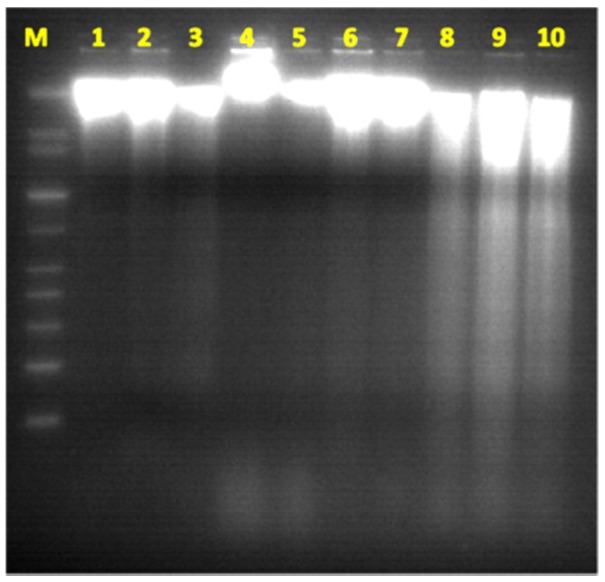
DNA-ladder fragmentation. The genomic DNA extracted from hepatic tissues showed a very weakly stained smear pattern upon electrophoresis, with no evidence of DNA-ladder pattern except from the reference area (1–5). In contrast, the genomic DNA extracted from samples (6–10) of polluted area revealed internucleosomal fragmentation (ladder pattern) mixed with a smear-like pattern, which are classified as signs of apoptosis and necrosis.

In the hepatic tissues, Cd showed the largest fold increase, while Zn had the lowest fold change in samples from the polluted area in comparison with that from the reference area. Cd may enter the atmosphere from mining, smelting, refining, and manufacturing processes. Excess Cd can cause serious damage to the brain, kidneys, nervous system and red blood cells. Young children, infants and fetuses are particularly susceptible to Cd poisoning compared to adults. Recent research has indicated that Cd may be implicated in causing various types of cancer, including renal [32], breast [33], pancreas [34], endometrial, and ovarian [35] and laryngeal and nasopharyngeal cancer [36]. Cadmium exposure has been associated with increased cancer risk, and low Zn levels appears to reduce exacerbate Cd carcinogenicity. Lin, *et al.* [37] demonstrated that Cd exposure is an important independent risk factor of cancer mortality in older Americans with low Zn intake. There are multiple molecular mechanism by which Cd induce cancer and the most important among them are aberrant gene expression, inhibition of DNA damage repair and induction of apoptosis [38,39]. Apoptosis or programmed cell death is a unique form of cell death that is associated with mechanism of morphogenesis and the normal cell turnover of adult tissues. It is recognized by its characteristic cellular morphology, particularly chromatin condensation in the nucleus and plasma membrane blebbing [40]. Later, it was found that apoptotic cells undergo internucleosomal cleavage of DNA forming a “ladder” pattern on agarose gel electrophoresis [41]. These morphological and biochemical features are accepted to be important criteria for confirmation of apoptosis. Previous research indicated that activation of endogenous endonuclease by cadmium result in DNA degradation, morphological and biochemical alterations seen in apoptosis [42]. In agreement with these data, analysis of DNA in our study revealed that hepatic tissues of fish samples from polluted area have significant increase in apoptotic cells as detected by flow cytometry (Figure 2) and formation DNA-ladder (Figure 3) in comparison to samples from reference area. These data suggest that Cd may play a role in these findings.

The present study also showed that the liver of fish samples from the polluted area contain significantly higher concentrations of Cr and Cu in comparison to those from the reference area. The Cr values detected in this study in both the reference and polluted areas were above the FAO limiting standards of 0.15 μg/g for food fish [43].

The WHO has proposed that Cr is a human carcinogen. Several studies have shown that chromium (VI) compounds can increase in risk of lung cancer [44,45]. The copper contents in the samples were also higher than the FAO-permitted level in fishes (3.0 μg/g). Excessive intake of copper may lead to liver cirrhosis. Hepatic copper accumulation occurs in a number of cholestatic diseases and they play an important part in pathogenesis and can occasionally lead to neurological toxic effects. Copper overload in the new-born period when biliary excretion of copper is inefficient may establish a vicious cycle of copper accumulation and liver damage [46].

In addition to copper, research indicate that iron deposition causes progressive liver cell damage and is involved the pathogenesis of liver cirrhosis and fibrosis [47]. Interestingly, Fe levels presented the second largest increase in samples from the polluted area. Iron is an essential element involved in various biological processes including oxygen transport, electron transport and various enzymatic reactions such as DNA synthesis, transcriptional regulation, catalysis as well as nitric oxide (NO) and oxygen sensing [48]. Several research studies demonstrated that deregulated cellular and systemic iron metabolism would lead to cytotoxicity and increased risk for various diseases, including nephrotoxicity and renal cancer [49,50]. In addition, it was demonstrated that iron induced tissue lipid peroxidation, apoptosis, and formation of 8-hydroxydeoxyguanosine, a known DNA oxidative modification [50]. 

**Figure 4:**
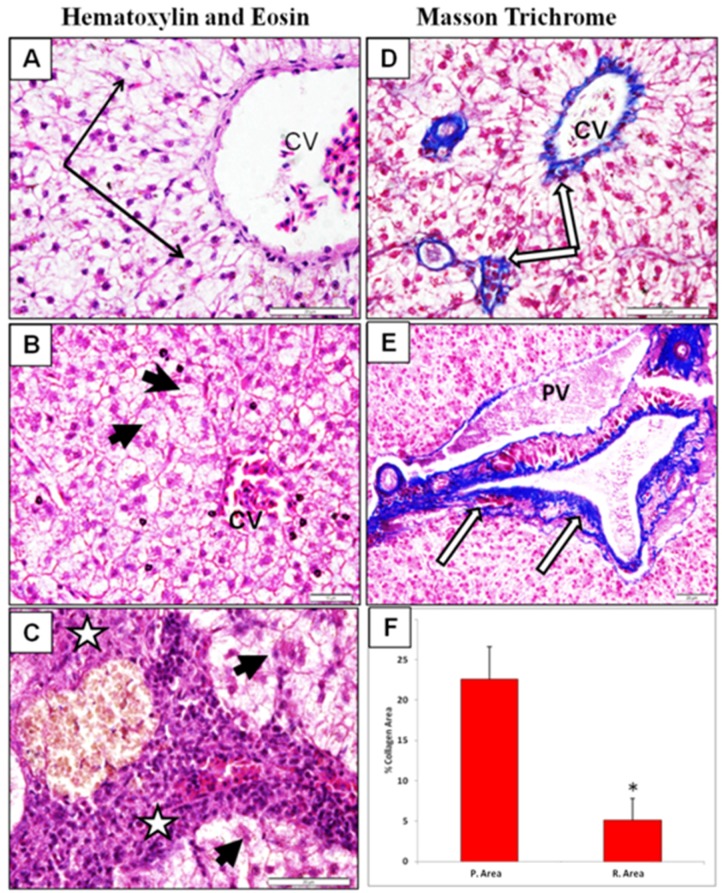
Liver sections stained with H&E (**A**–**C**) and Masson trichrome for collagen (**D**,**E**); (**A**) Sections of fish from reference area exhibiting normal hepatocyte cell cords around the central vein (CV) (black arrows). In polluted group (**B**,**C**) there is loss of normal organization and marked fibrosis and inflammatory cell infiltrate (stars) with swollen hepatocytes filled with lipid droplets (arrow head); (**D**) liver section from reference area stained with Masson trichrome showing moderate increase in collagen fiber deposition around the CV (white arrows); (**E**) liver sample from polluted area near portal vein region showing marked increase in collagen fiber deposition. Notice the dark staining of hepatocytes in polluted group (white arrow); (**F**) Quantitative assessment of collagen percentage using ImageJ software as described in methods. Values represent the means ± SD, * *p* < 0.05 (Student *t*-test).

In mammals, iron overload, especially at sites of storage such as the liver, enhances oxidative stress leading to lipid, nucleic acid and protein peroxidation. In the liver, lipid peroxidation results in damage to hepatocellular organelles, such as mitochondria and lysosomes, which is thought to contribute to hepatocyte necrosis and apoptosis, and ultimately lead to the development of hepatic fibrogenesis [51]. Fibrosis is associated with major quantitative and qualitative changes in the extracellular matrix including increased expression of fibrillary collagens I and III, collagen IV, fibronectin, elastin and laminin [52,53]. To test if the increased content of iron in our samples may associates with liver fibrosis, we examined hepatic sections for fibrotic changes and collagen deposition by hematoxylin-eosin staining and Masson’s trichrome staining, respectively. The intensity of Masson’s trichrome blue color staining depended on the content of collagen fibers in the investigated tissue [27]. We found that liver sections from polluted area exhibited significant fibrotic alterations and increased percentage of collagen stained area in comparison to samples taken from reference area (Figure 4).

**Figure 5:**
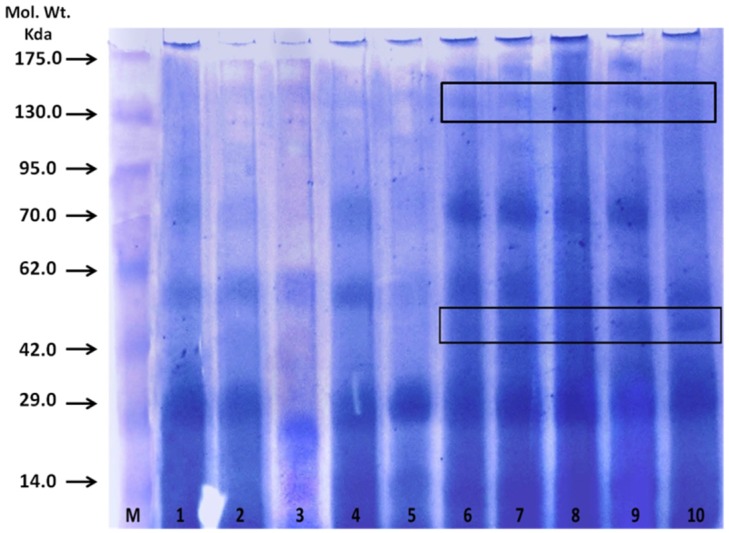
The electrophoretic patterns of proteins of liver of *Variola louti* fish caught in the reference (1–5) and polluted areas (6–10).

During liver fibrosis, the liver total collagen content is increased by four- to seven-fold, in particular type I and type III and IV collagens [54]. In the present study, electrophoretic patterns of proteins of liver of *Variola louti* fish caught in the polluted area showed similar protein patterns as the reference area, except for some protein bands that appeared in polluted area fish (Figure 5). Protein bands of 50 kDa appeared in five samples from the polluted area (lanes 6–10). Unusual protein bands situated at 130 and 140 kDa were also detected in polluted area samples, which is in a good agreement with the molecular weight of collagen type III. In a previous investigation, Mohamed [55] attributed pathological alterations in liver of *Oreochromis niloticus* and *Lates niloticus* from Lake Nasser, Egypt to the cumulative effect of metals and the increase in their concentrations in the liver.

## 4. Conclusions

In conclusion, heavy metals in polluted water have an impact on the DNA and protein of fishes taken from this polluted water. Thus, ingestion of contaminated seafoods should receive special attention in order to protect human beings from these dangerous hazards. Assessment of fishes may provide a tool to monitor risks to general health of human and wild life.

## Abbreviations

The following abbreviations are used in this manuscript:

R. Area	Reference area
P. Area	Polluted area
CV	central vein
OD	optic density
FL-A	peaks area
FL-W	peaks width
FCM	flow cytometry
